# Inter- and intra-rater variability of MRI-based lesion size measurements in active surveillance for prostate cancer: a multicentre study

**DOI:** 10.1007/s00330-025-12318-1

**Published:** 2026-02-06

**Authors:** Cameron Englman, Busola Adebusoye, Michele Cosenza, Andrea Del Prete, Louise Dickinson, Giulio Imperiale, Riccardo Leni, Giorgio Gandaglia, Francesco De Cobelli, Sue Mallett, Alex Kirkham, Caroline M. Moore, Francesco Giganti, Giorgio Brembilla

**Affiliations:** 1 Division of Surgery & Interventional Science, University College London, London, UK; 2 Department of Radiology, University College London Hospitals NHS Foundation Trust, London, UK; 3 Centre of Medical Imaging, Division of Medicine, University College London, London, UK; 4 Department of Radiology, IRCCS San Raffaele Scientific Institute, Milan, Italy; 5 Vita-Salute San Raffaele University, Milan, Italy; 6 Division of Experimental Oncology, Department of Urology, IRCCS San Raffaele Scientific Institute, Milan, Italy; 7 Department of Urology, University College London Hospitals NHS Foundation Trust, London, UK

**Keywords:** Prostate cancer, Prostate magnetic resonance imaging, Active surveillance, Measurement reliability

## Abstract

**Objectives:**

Prostate cancer (PCa) lesions can be measured on MRI using maximum or biaxial diameters, or as volumes derived by the ellipsoid formula or planimetry. We evaluated the inter- and intra-rater reliability (reliability between different radiologists and the same radiologist during different reading sessions) of lesion size measurements on baseline MRI scans for patients on active surveillance (AS).

**Materials and methods:**

Twenty patients with low- or intermediate-risk PCa (Gleason score 3 + 3 or 3 + 4) and MRI-visible lesions were selected from AS cohorts at two centres (United Kingdom and Italy). Five radiologists, blinded to clinical outcomes and reports, independently measured the index lesion on a baseline MRI scan in a single reading session using: (1) maximum diameter; (2) biaxial diameters; (3) ellipsoid volume, and (4) planimetry volume. Measurements were repeated after a 4-week washout period. Strip plots present lesion size measurements for all methods and readers. Bland–Altman plots were used to present intra-rater reliability.

**Results:**

Graphical presentation of measurements across the twenty patients enabled examination of variability between methods, readers, and reads. There was considerable variation for all methods, and for a single lesion, size measurements spanned previously accepted definitions of clinically significant and insignificant disease. Inter-rater reliability decreased for larger lesions, with notable radiologist-specific differences, and intra-rater reliability appeared better overall.

**Conclusion:**

This study underscores the difficulty of reliably measuring PCa lesions during AS. Intra-rater reliability appeared greater than inter-rater reliability, emphasising that radiologists should remeasure lesions when tracking changes. More work is needed on measuring change in lesion size across serial MRI scans.

**Key Points:**

***Question**** Several methods exist for measuring prostate cancer lesion size on MRI for patients on active surveillance, but it is unclear how reliable these methods are*.

***Findings**** Lesion size measurements varied widely across methods and readers, often spanning thresholds for clinically significant disease, and intra-rater reliability was generally better than inter-rater reliability*.

***Clinical relevance**** Variability in lesion size measurements on MRI may lead to inconsistent clinical decisions during active surveillance. Our findings emphasise that regardless of the method used, lesions should be remeasured by the same radiologist when monitoring patients on active surveillance*.

**Graphical Abstract:**

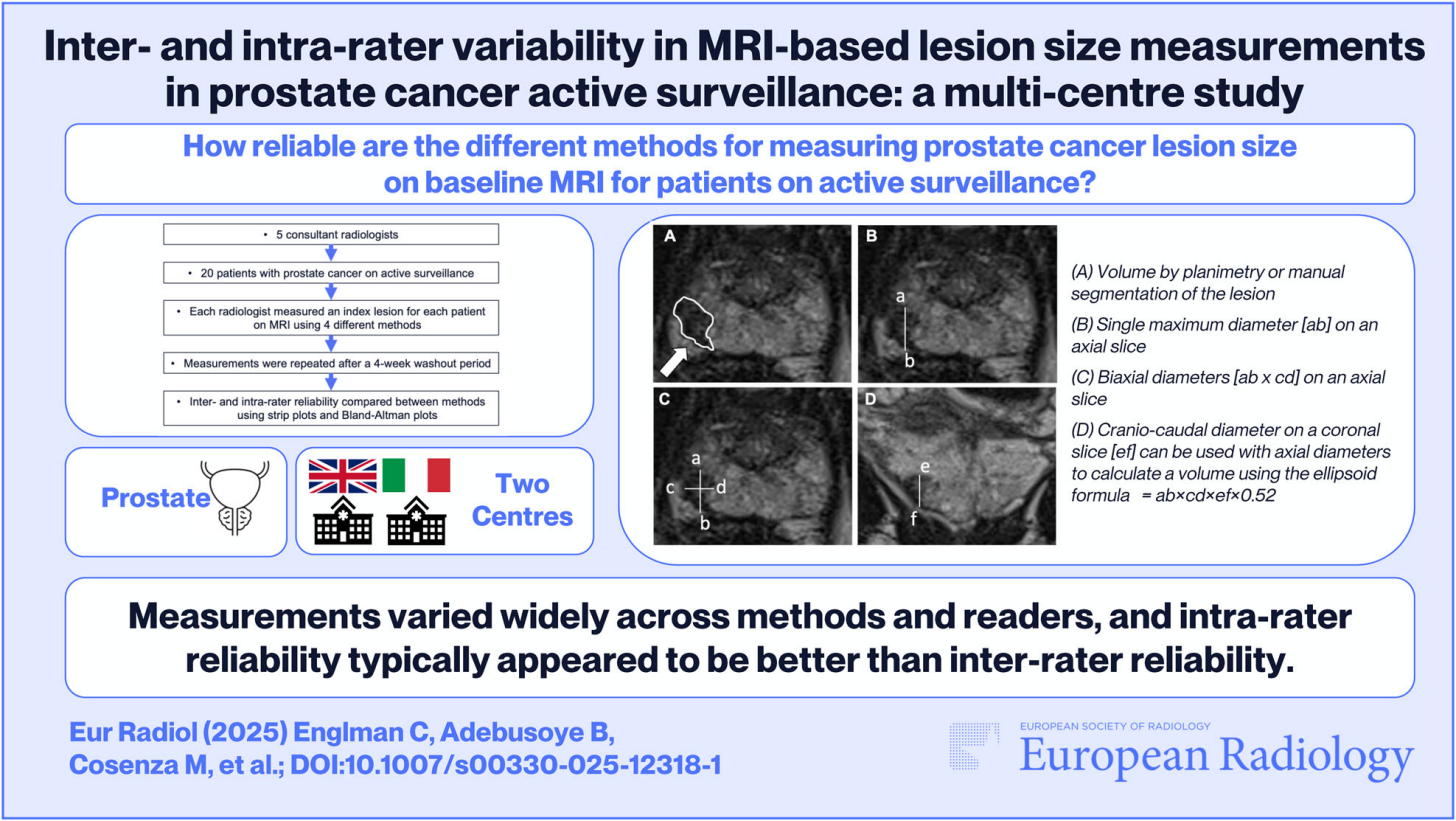

## Introduction

Active surveillance (AS) is a management option for patients with low- to intermediate-risk prostate cancer (PCa). It involves close monitoring to defer or avoid treatment, thereby reducing over-treatment and treatment-related side effects, while still maintaining the opportunity for curative treatment if the tumour progresses or a patient chooses. AS protocols typically consist of a combination of serial prostate-specific antigen (PSA) tests, prostate biopsies, and multiparametric MRI scans [1–3].

The visibility of a lesion on MRI at baseline is a significant risk factor for PCa progression [4, 5], and although PCa is often multifocal, growing evidence suggests that it is primarily the most prominent tumour focus, known as the index lesion, that drives disease progression and impacts oncological outcomes [6–9]. While the size of the index lesion on MRI is also considered important, a 1.5 cm diameter threshold forms the cut-off between a score of 4 and 5 in the Prostate Imaging-Reporting and Data System (PI-RADS) v2.1.

**Fig. 1:**
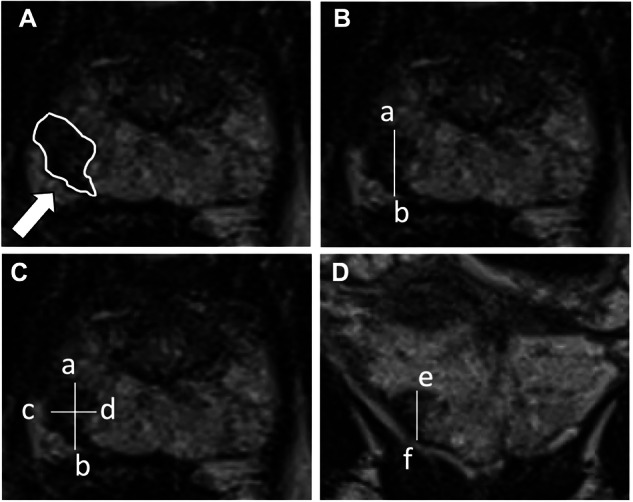
Commonly used methods to measure prostate cancer lesion size. A 1.5-T scan of a 56-year-old patient with 5 mm GS 3 + 3 in the right peripheral zone on targeted biopsy. The lesion between 7 and 9 o’clock (arrow) is clearly visible on the axial *T2-*WI. **A** The volume of the prostate cancer lesion indicated by the white arrow can be measured by tracing around the lesion on each axial slice, often referred to as manual segmentation or planimetry. **B** A single maximum diameter [ab] can be used to measure size. **C** Biaxial diameters [ab × cd] are often used to measure size. **D** The craniocaudal diameter on a coronal image [ef], along with the axial diameters, can be used in the ellipsoid formula to measure lesion volume [Volume = ab × cd × ef × 0.52]. GS, Gleason Score

Methods for measuring PCa lesion size on MRI include: (1) single maximum diameter; (2) biaxial diameters; (3) volume by ellipsoid formula and (4) volume by planimetry (Fig. 1). While maximum diameter is the quickest and most widely used method, biaxial and ellipsoid measurements provide additional detail, and planimetry offers the most comprehensive assessment of lesion size, albeit at the cost of greater time and effort. However, there is no consensus on the best method for measuring and assessing lesion size, as also outlined in the Prostate Cancer Radiological Estimation of Change in Sequential Evaluation (PRECISE) v2 recommendations [10, 11].

To help address this issue, we aimed to determine the inter- and intra-rater reliability of different methods for measuring PCa lesion size on the baseline MRI scan of patients on AS.

## Materials and methods

### Study design

Clinical records and MR images are routinely reviewed as part of an audit performed for service evaluation of the AS programme at each centre and therefore no institutional review board or research ethics committee approval was required.

### Patient population

Twenty eligible patients with low- or intermediate-risk PCa (Gleason score (GS) 3 + 3 or 3 + 4) were randomly filtered out from retrospectively maintained AS databases at two centres: University College London Hospitals (UCLH), London, United Kingdom and San Raffaele Hospital (OSR), Milan, Italy.

To be eligible for inclusion, patients were required to have a focal lesion on baseline MRI (Likert or PI-RADS v2.1 4–5) and at least two follow-up scans. Scans of suboptimal diagnostic quality (Prostate Image Quality (PI-QUAL) v1 score 1 to 3 [12]), or where the focal lesion could not be clearly delineated, were excluded.

Full inclusion and exclusion criteria are in Table 1. Supplementary Figure S1 demonstrates a patient inclusion flow diagram.

**Table 1:** Patient inclusion and exclusion criteria

Inclusion criteria	Exclusion criteria
•Patients must be on MRI-influenced AS at UCLH or OSR	•Patients with only 1 or 2 mpMRI scans
•Patients with at least 3 mpMRI scans (1 baseline scan and 2 follow-up scans)	•Patients with consecutive scans less than 1 year apart or more than 5 years apart
•Follow-up scans over a year apart (in keeping with the PRECISE v2 recommendations)	•MRI scans that are not optimal quality (PI-QUAL v1 1–3)
•At least 3 MRI scans of high-quality (PI-QUAL v1 4 or 5)	•Patients with diffuse lesions or no MRI-visible lesions (PI-RADS 1–3)
•Patients with a clearly visible focal/discrete lesions (PI-RADS 4 or 5) in the PZ or TZ across multiple scans	•Patients with a focal/discrete lesion recorded on only 1 MRI scan
•Patients with an MRI and biopsy with concordant results (where MRI and biopsy findings agree on location and risk level of cancer)	•Patients with biopsy results that are not concordant with MRI findings

*mpMRI* multiparametric magnetic resonance imaging, *OSR* Ospedale San Raffaele, *PI-QUAL* prostate imaging quality, *PI-RADS* Prostate Imaging-Reporting and Data System, *PRECISE* Prostate Cancer Radiological Estimation of Change in Sequential Evaluation, *PZ* peripheral zone, *TZ* transition zone, *UCLH* University College London Hospitals

### MRI protocol

At UCLH, seven different MRI scanners were included in the analysis: four 1.5-Tesla scanners (Siemens) and three 3-Tesla scanners (one Siemens and two Philips). At OSR, two MRI scanners were used: two 1.5-Tesla scanners (Philips). Standard multiparametric MRI sequences were acquired (Table S1).

Baseline prostate MRI scans for each patient were anonymised and uploaded to an online cloud-based viewing and storage platform (MIMcloud®).

### Image analysis

Five consultant radiologists from the two centres (three OSR and two UCLH), with varying experience (3–20 years) in reporting prostate MRI, independently assessed all scans. They were blinded to any previous clinical or radiological information, and to the measurements from other readers.

**Fig. 2:**
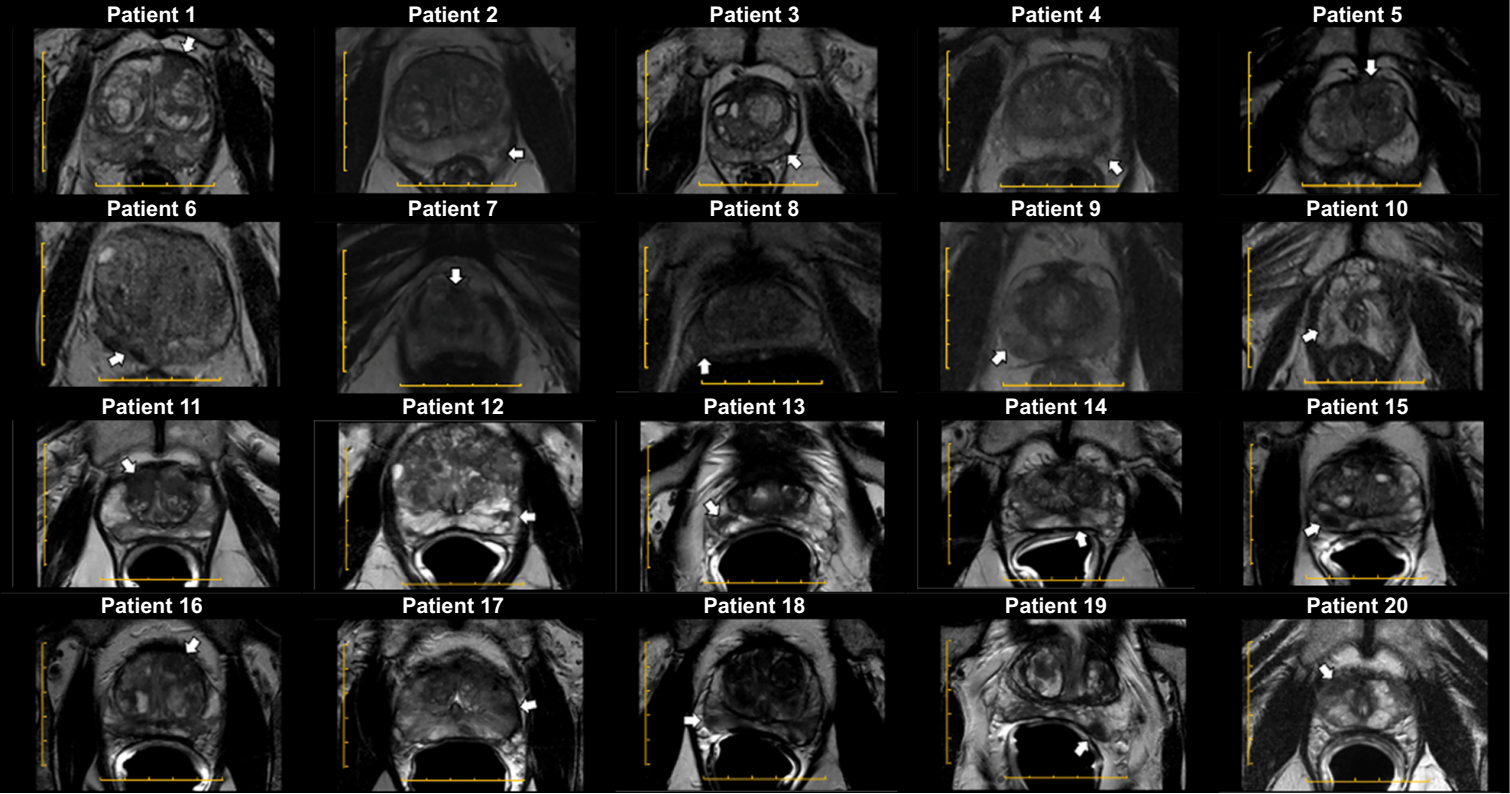
Prostate cancer lesions at baseline on the T2-weighted magnetic resonance image from patients on active surveillance. Prostate cancer lesions on the T2-WI from patients on active surveillance at University College London Hospitals (Patients 1–10) and Ospedale San Raffaele (Patients 11–20). White arrows point to the lesion of interest (the index lesion) for each subject, and the scale in each scan is 5 mm in length

An index lesion on the baseline MRI for each patient initially identified during routine clinical reporting was pointed out to the radiologists on a PowerPoint slide before size measurements (Fig. 2). The radiologists were given written instructions directing them to measure the maximum diameter of the lesion on the axial slice, biaxial diameters on the axial slice (the maximum anterior-posterior and transverse diameter), as well as the ellipsoid formula and the volume by planimetry for each scan were measured on T2-weighted images (T2-WI), with other MRI sequences used to aid in identification and measurement of margins, as per the PRECISE v2 recommendations [11].

All readers repeated the lesion size measurements in a second reading session following a 4-week washout period to assess intra-reader reliability.

### Statistical analysis

Summary statistics were used for patient characteristics. Medians and quartiles were reported for continuous variables, while frequencies and proportions were reported for categorical variables.

The different methods of measuring lesion size were compared with biaxial diameters multiplied together to form a single number (providing an estimated surface area on the largest slice).

Absolute values for lesion size were displayed as strip plots comparing radiologists for the different methods of lesion measurement. Patients were arranged by the median lesion diameter to illustrate how size influences measurement variability.

Quantitative thresholds for lesion size associated with clinically significant PCa, as previously reported in the literature, were also indicated on the strip plots along with their corresponding references.

Bland–Altman plots were used to visualise the inter-rater agreement between the first and second reading sessions for each method of lesion measurement.

Intraclass correlation coefficients (ICC) were calculated and reported along with the 95% confidence intervals (CI). The intra-rater and inter-rater reliability coefficients were calculated using the random factorial design where both the subjects and the raters are randomly selected from the respective populations they represent to yield a result based on two-way random effects analysis of variance (ICC [1, 2]), as previously described [13].

Statistical analyses were performed using R (R Foundation for Statistical Computing; URL: https://www.R-project.org/), and the Guidelines for Reporting Reliability and Agreement Studies (GRRAS) [14] were followed.

## Results

### Study sample

The cohort consisted of twenty patients, who had enrolled on AS programmes from April 2013 to June 2021, half of whom had GS 3 + 4 disease, and the majority (70%) had an index lesion in the peripheral zone (PZ). Table 2 illustrates the patient demographics and PCa characteristics for the sample population.

**Table 2:** Patient and prostate cancer lesion characteristics for the sample population

	UCLH	OSR
Number of patients	10	10
Age at diagnosis, median (Q_1_, Q_3_)	65 (60, 71)	62 (58, 67)
Presenting PSA, median (Q_1_, Q_3_)	5.5 (4.9, 7.8)	5.4 (4.4, 7.4)
GS on baseline biopsy		
3 + 3 (%)	0	10 (100)
3 + 4 (%)	10 (100)	0
MCCL, median (Q_1_, Q_3_)	5 (4, 6)	*
Index lesion location		
PZ (%)	7 (70)	7 (70)
TZ (%)	3 (30)	3 (30)
Index lesion side		
Right (%)	4 (40)	5 (50)
Left (%)	5 (50)	5 (50)
Midline (%)	1 (10)	0

*GS* Gleason score, *MCCL* maximum cancer core length, *OSR* Ospedale San Raffaele, *PSA* prostate-specific antigen, *PZ* peripheral zone, *Q* quartile, *TZ* transition zone, *UCLH* University College London Hospitals

* Not regularly reported on prostate biopsy histopathology results

### Inter-rater reliability

**Fig. 3:**
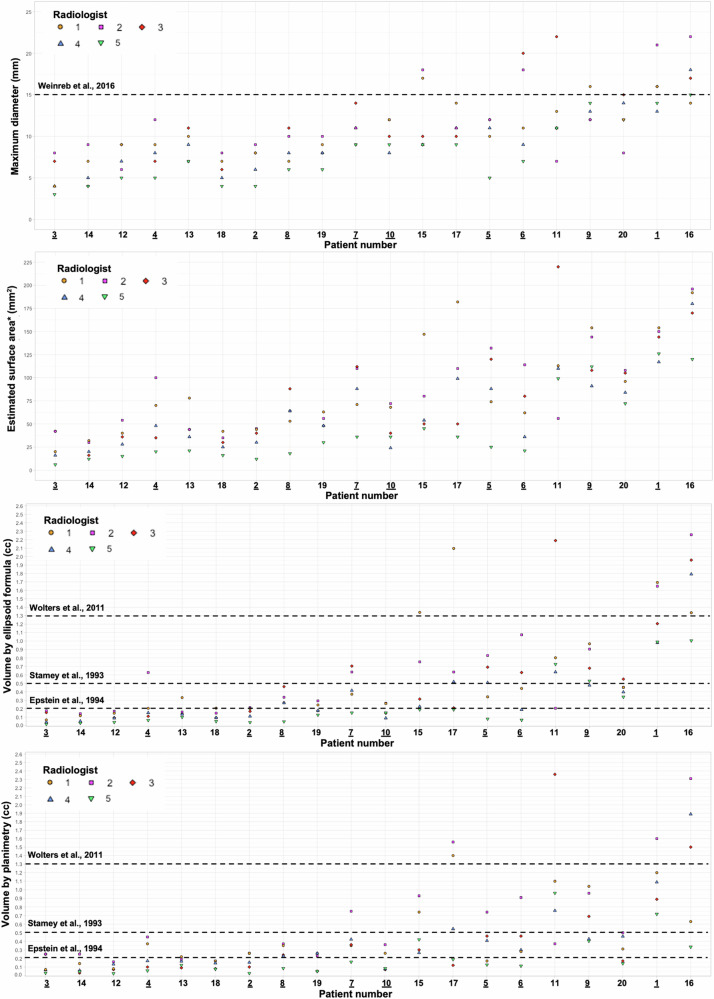
Strip plots with the measurements for each method from the first reading session. Patients are arranged by the median lesion diameter, with the smallest on the left, and patients with Gleason 3 + 4 disease are underlined. Dotted lines represent previously established thresholds for clinically significant prostate cancer lesion size, along with the associated reference above them. * Estimated surface area calculated by the multiplication of biaxial diameters on the axial slice

Figure 3 shows strip plots of lesion size measurements from the first reading session. There was considerable variation in lesion size measurements among radiologists for all methods. For a single lesion, lesion size measurements spanned previously accepted definitions of clinically significant and insignificant disease, and the spread of radiologists across boundaries that indicate clinical significance varied. In the first reading session, 7/20 patients had maximum diameter measurements that, across different radiologists, spanned the proposed 1.5 cm threshold for clinical significance, which is also used to distinguish between a PI-RADS 4 and 5 lesion. For volume by the ellipsoid formula, 5/20 patients had measurements spanning the 1.3 cc clinical significance threshold, while for planimetry, this was 4/20 patients. Additionally, 8/20 patients had measurements that spanned the 0.5 cc volume threshold when measured by different radiologists using the ellipsoid formula and planimetry.

There were also systematic differences in measurement tendencies. Certain radiologists consistently recorded higher or lower measurements across all methods. For instance, Radiologist 2 frequently reported the highest measurements for maximum diameter, biaxial diameters, and volume metrics.

### Intra-rater reliability

**Fig. 4:**
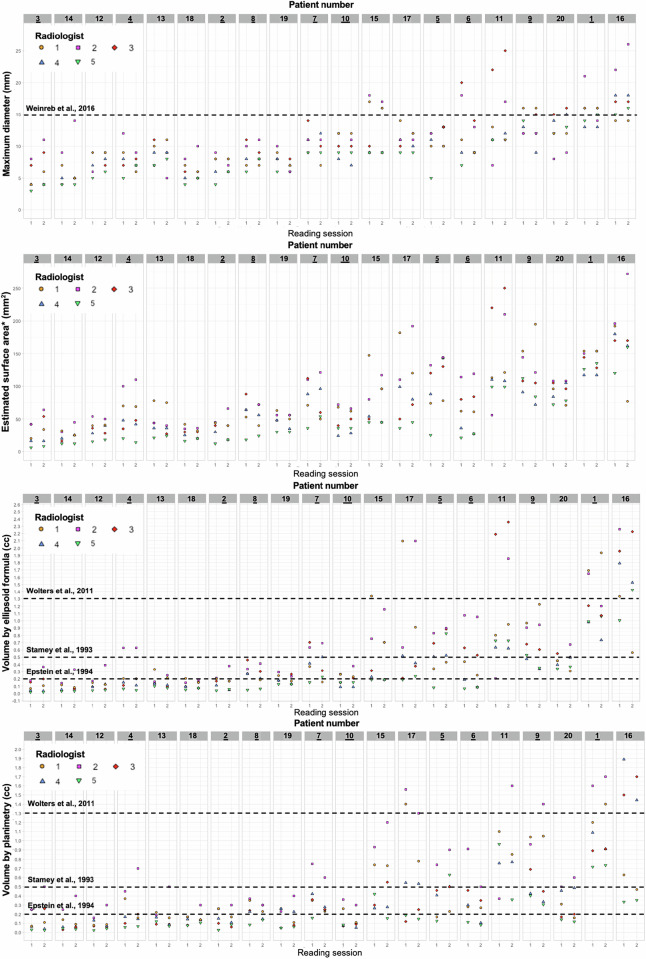
Strip plots with the measurements for each method from both reading sessions. Patients are arranged by the median lesion diameter, with the smallest on the left, and patients with Gleason 3 + 4 disease are underlined. Dotted lines represent previously established thresholds for clinically significant prostate cancer lesion size, along with the associated reference above them. * Estimated surface area calculated by the multiplication of biaxial diameters on the axial slice

Figure 4 presents strip plots for each method of measurement for both reading sessions. Visual inspection of these graphs demonstrates that the reliability between the same readers across two different reading sessions appears to be higher than the reliability between different readers. However, there were instances where an individual radiologist’s measurements crossed thresholds for clinical significance across the two reading sessions, with some lesions classified as significant in one reading but not in the other.

**Fig. 5:**
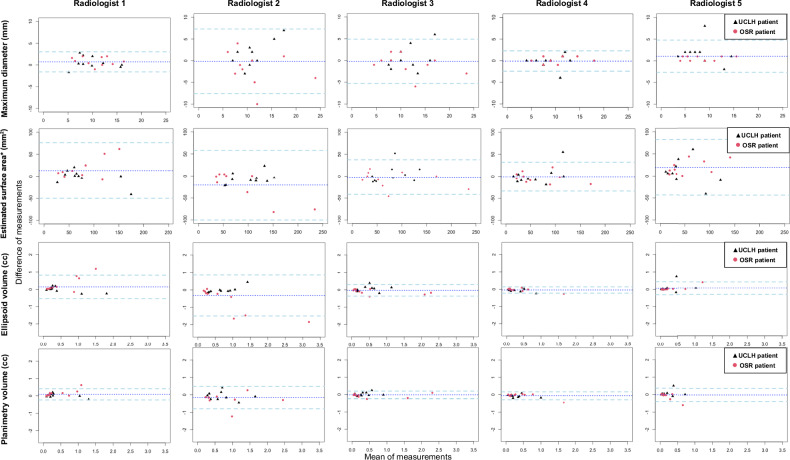
Bland–Altman plots demonstrating levels of agreement between the first and second read for each method of measuring lesion size for all reporters stratified by outliers. Lesions from patients at University College London Hospitals (UCLH) with Gleason 3 + 4 disease (▴) and patients at San Raffaele Hospital (OSR) with Gleason 3 + 3 disease (⚫) are indicated

In Bland–Altman plots (Fig. 5), across all methods, most data points clustered around the zero-difference line, indicating overall agreement between reading sessions. There was variability amongst radiologists, with Radiologists 3 and 4 showing the greatest consistency. On visual inspection, we found no clear relationship between the experience level of the radiologists and the intra-rater reliability. Measurements were also stratified by patient centre, and therefore by Gleason score (since all OSR patients had GS 3 + 3 disease and all UCLH patients had GS 3 + 4), but this stratification did not appear to affect the levels of agreement.

The ICC values for intra-rater and inter-rater reliability are shown in Table 3. These were also consistently higher for intra-rater reliability when compared to inter-rater reliability.

**Table 3:** Intraclass correlation coefficients for the inter-rater and intra-rater measurement reliability

Method of measurement	Inter-rater reliability (1st reading session) ICC (95% CI)	Inter-rater reliability (2nd reading session) ICC (95% CI)	Intra-rater reliability ICC (95% CI)
Maximum diameter	0.54 (0.33–0.75)	0.58 (0.38–0.77)	0.83 (0.76–0.88)
Biaxial diameters	0.63 (0.41–0.81)	0.61 (0.38–0.79)	0.82 (0.74–0.87)
Ellipsoid volume	0.59 (0.39–0.78)	0.52 (0.31–0.73)	0.77 (0.68–0.84)
Planimetry volume	0.51 (0.31–0.72)	0.49 (0.26–0.72)	0.90 (0.86–0.93)

*CI* confidence interval, *ICC* intraclass correlation coefficient

## Discussion

We addressed an important clinical question regarding the reliability of different methods for measuring lesion size, a topic that is fundamental to the use of MRI in AS, but for which there is currently a gap in the literature.

The PRECISE v2 consensus panel could not reach an agreement on the best method for measuring lesion size during AS, and polling the 20 specialist prostate radiologists on the panel indicated that 25% report lesion volume in clinical practice (15% by planimetry and 10% by ellipsoid), 40% report lesion size using a single diameter, and 30% report the two axes diameter [11].

### Graphical presentation of individual measurements

The focus of presenting results for this study has been to show the individual results, by lesion, by measurement method, repeat readings, and by reader. A huge amount of information and important insights can be gained from the visual inspection of the individual data as presented on the strip plots.

Although ICC results are typically included in reliability studies and are reported, we have deliberately avoided drawing conclusions based on these values as they can be misleading summaries [15, 16]. The ICC values average across all lesions without accounting for individual lesion characteristics that may influence measurement reliability. As such, ICC summaries are highly dependent on the prevalence of lesions that can be measured reliably or less reliably, rather than providing insights specific to lesion characteristics or different methods of size measurement. For these reasons, our discussion prioritises results from the interpretation of the graphical data over ICC values.

### Summary of inter- and intra-rater reliability results

We observed considerable variation in lesion size measurements between radiologists across all methods, with measurements spanning previously accepted definitions of clinically significant disease (Fig. 3). This finding raises important concerns about the role of quantitative size thresholds guiding clinical decision-making, given the substantial variability in lesion measurements.

Overall, as supported by both the strip plots and ICC values, the measurement variability between different readers is greater than the variability observed when the same reader repeats measurements after a 4-week washout period (Fig. 4). This suggests that while individual radiologists measure lesions relatively consistently over time, more substantial differences exist between radiologists.

Bland–Altman plots (Fig. 5) allowed us to assess differences in measurements between first and second readings for each radiologist. For maximum diameter and biaxial diameters, there was large measurement variability between readings for some patients, reflecting the challenges even with these simpler measurement methods for some lesions. In contrast, volume-based methods, both the ellipsoid volume and planimetry, appeared to display narrower limits of agreement, with planimetry performing slightly better overall. Additionally, we found no evidence that intra-rater reliability was influenced by patient centre, challenging the common assumption that radiologists perform better when interpreting scans from their own institution.

### Potential explanations for measurement discrepancies

**Fig. 6:**
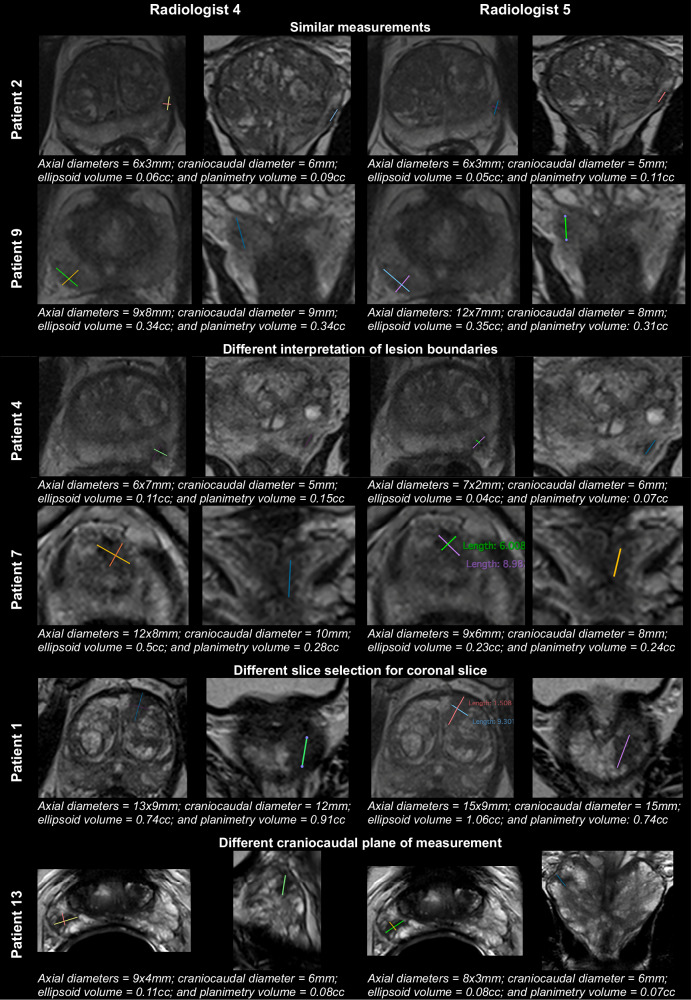
Potential reasons to explain measurement discrepancies between radiologists

Radiologists 4 and 5 captured screenshots of their diameter measurements during the second reading session. Selected examples, presented in Fig. 6, illustrate several possible reasons for discrepancies in lesion size measurements between readers. These include differences in the interpretation of lesion boundaries, variation in slice selection, and measurements taken on different craniocaudal planes. Beyond written instructions to measure lesion size using the different methods, no formal training session was conducted, which may have contributed to these inconsistencies. These findings emphasise the importance of standardised measurement protocols, as well as training and calibration exercises for radiologists, and, where feasible, the same-radiologist follow-up to minimise variability.

### Clinical implications

This study highlights that lesion size measurements are less straightforward and more variable than commonly assumed. The strip plots revealed that measurements frequently spanned established thresholds for clinically significant disease, not only between different radiologists but also, at times, by the same radiologist across separate readings. Such variability can lead to inconsistent assessments of disease stability or progression, potentially resulting in misinformed treatment decisions.

Radiologists should be mindful of how their measurements can influence clinical decisions, particularly since some urologists may rely on specific size thresholds to guide biopsy or treatment decisions, although this is an area that remains largely unexplored in the literature. Equally, urologists should account for potential inter-rater variability when interpreting lesion measurements reported by different radiologists and should be aware that reliance on single or threshold-based measurements for management decisions is risky due to high inter- and intra-rater variability.

The findings also emphasise the importance of consistency in measurement methods. For accurate assessments of lesion size across time, urologists should ideally rely on lesion size measurements made using the same method and, where possible, by the same radiologist. In line with PRECISE v2 recommendations [11], radiologists are encouraged to remeasure lesions on prior scans when interpreting a new follow-up scan, rather than relying solely on previous reports, whether authored by themselves or others. This also raises an important practical question about how radiologists should address and report discrepancies when their current measurements differ from earlier assessments.

### Comparison with existing literature

Reliable and consistent lesion size measurements are critical for monitoring change during AS and may also help identify patients suitable for AS. Despite this importance, few studies have directly assessed the reliability of different measurement methods in PCa. To our knowledge, this is the first study to compare intra- and inter-reader reliability across multiple approaches in the AS setting.

Histological studies have long sought to define thresholds for clinically significant disease. Stamey et al [17] examined 139 cystoprostatectomy specimens from bladder cancer patients and reported incidental PCa in 40% of cases. Based on tumour volume distribution, they estimated that lesions larger than 0.5 mL were clinically significant. Epstein et al [18] subsequently categorised radical prostatectomy specimens as insignificant (< 0.2 cm³), minimal (0.2–0.5 cm³), or moderate (> 0.5 cm³). Similarly, Wolters et al [19] analysed 325 prostatectomy specimens from the European Randomised Study of Screening for Prostate Cancer, identifying thresholds of 0.55 mL for insignificant disease and 1.3 mL for patients with Gleason 3 + 3 tumours. These findings informed the adoption of a 1.5 cm MRI lesion diameter cut-off in PI-RADS v2 [20, 21].

Several MRI-based studies have assessed measurement reliability. Although these have generally relied on ICC summary values, rather than presenting graphical data where differences between lesions can be distinguished. Diaz de Leon et al [22] asked four readers to measure maximum lesion diameter in 80 men with localised PCa, reporting inter-reader ICC of 0.74 (95% CI 0.66–0.82) and intra-reader ICC of 0.91 (0.87–0.94). Harvey et al [23] compared tumour volume across two readers on individual MRI sequences and against whole-mount histopathology, finding the highest accuracy and reliability on T2-WI.

There have also been some direct comparisons of MRI-derived lesion size with histopathology from radical prostatectomy specimens [22, 24–30] or template mapping biopsies [31], using both maximum diameter [22, 26–28] and tumour volume estimated by planimetry [24, 25, 31] or the ellipsoid formula [29, 30]. Marin et al [32] evaluated manual, semi-automated, and volumetric methods, showing that maximum diameter correlated most strongly with histological measurements, while ellipsoid and planimetric volumes demonstrated good inter-reader agreement. Nonetheless, other studies report variability in correlations between measurements on MRI and histology for tumour volume [33]. Pooli et al [34] found MRI often underestimates pathological tumour size, particularly for small or low PI-RADS v2 lesions. It has been observed that discrepancies between lesion size on MRI and histopathology may arise from prostate sectioning techniques [35, 36] or inconsistencies in histopathological slide preparation [33, 37].

Other studies on MRI during AS have examined both diameter [38–42] and volume measurements [43–48]. Our previous work confirmed a strong correlation between ellipsoid and planimetric methods [49]. Similar challenges in the assessment of lesion size have been encountered by pathologists [50], underscoring the broader need for measurement standardisation across both imaging and histopathology during AS.

### Limitations

Our study has some limitations, the first being the sample size. While some radiologists may have been willing to review more patients, we needed to carefully consider the workload required for the study and the number of scans per reporter to encourage multiple radiologists to participate. Second, although we found no evidence that our study findings were influenced by MRI scanner or the Gleason grade of enrolled patients, our scans were performed using different scanners and magnets across two centres with different AS protocols: UCLH currently enrols patients with mostly intermediate-risk disease (GS 3 + 4), while OSR enrols patients with mostly low-risk disease (GS 3 + 3). We acknowledge that local practice patterns may limit the generalisability of our findings. Furthermore, as our study included only patients with low- and intermediate-risk prostate cancer on AS, the results may not be directly applicable to those with high-risk disease.

### Future directions

Future research should evaluate lesion size measurements across serial MRI scans to clarify whether absolute values or percentage changes best capture progression during AS. PRECISE v2 recommends T2-WI for primary measurement [11], whereas PI-RADS v2.1 suggests apparent diffusion coefficient maps for PZ lesions and T2-WI for transition zone lesions [51]. Determining the optimal sequence, especially for indistinct lesions, could improve accuracy and reliability.

Given the high variability observed across methods, standardised protocols are needed. The scan dataset from this study could serve as training and audit material to benchmark radiologist performance. Our findings of substantial inter-reader variability, with measurements frequently crossing thresholds for clinically significant disease, reinforce the need for further research to refine the definition of clinically significant lesions on MRI and to establish clear criteria for when changes in lesion size should prompt interventions such as biopsy or treatment.

Finally, artificial intelligence (AI)-based segmentation warrants investigation. Future studies should compare the reliability of AI-derived measurements with expert radiologists, given the rapid growth in adoption and technical capability [52, 53].

In conclusion, our study highlights the challenges of reliably measuring PCa lesions during AS. For individual MRI lesions, size measurements frequently spanned established thresholds distinguishing clinically significant from insignificant disease. We also observed that intra-rater reliability was generally higher than inter-rater reliability, emphasising the importance of radiologists remeasuring lesions themselves when assessing the changes in size over time during AS. To support informed decision-making and improve patient management in AS, future research should focus on serial lesion size measurements and enhancing measurement reliability through standardised protocols and sequence-specific analyses.

## Supplementary information

ELECTRONIC SUPPLEMENTARY MATERIAL

## Abbreviations

AI: Artificial intelligence
AS: Active surveillance
GS: Gleason score
ICC: Intraclass correlation coefficient
MRI: Magnetic resonance imaging
OSR: Ospedale San Raffaele/San Raffaele Hospital
PCa: Prostate cancer
PI-RADS: Prostate Imaging-Reporting and Data System
PRECISE: Prostate Cancer Radiological Estimation of Change in Sequential Evaluation
T2-WI: T2-weighted image
UCLH: University College London Hospitals
