# Supplementary material for: Inter- and intra-rater variability of MRI-based lesion size measurements in active surveillance for prostate cancer: a multicentre study
Source: Eur Radiol. 2026 Feb 6;36(7):5706–18. doi: 10.1007/s00330-025-12318-1 (PMC13282365; doi:10.1007/s00330-025-12318-1)
Supplement: Supplementary file 1 — ELECTRONIC SUPPLEMENTARY MATERIAL [file 330_2025_12318_MOESM1_ESM.pdf]

Inter- and intra-rater variability of MRI-based lesion size measurements in active surveillance for prostate cancer: a multi-centre study

ELECTRONIC SUPPLEMENTARY MATERIAL

Figures
Figure S1. Patient inclusion flow diagram

Tables
Table S1. Technical specifications for MRI scans

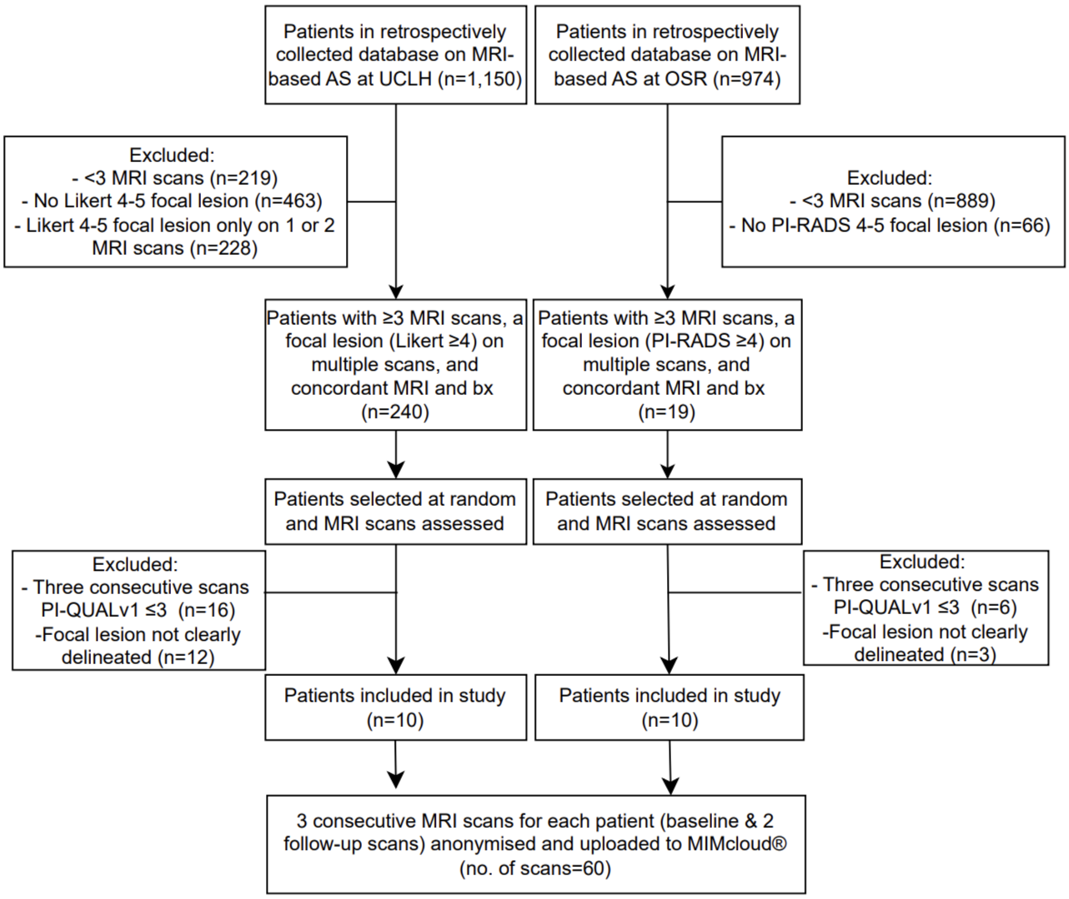

Figure S1. Patient inclusion flow diagram

Abbreviations: AS = active surveillance; bx = biopsy; mpMRI = multiparametric magnetic resonance imaging; n = number; no. = number; OSR = Ospedale San Raffaele; PI-QUAL = Prostate Imaging Quality; PI-RADS = Prostate Imaging–Reporting and Data System; PRECISE = Prostate Cancer Radiological Estimation of Change in Sequential Evaluation; UCLH = University College London Hospitals.

Table S1. Technical specifications for MRI scans

	UCLH		OSR
	**1.5T**	**3T**	**1.5T**
**T2-weighted imaging (axial)**			
Sequence	TSE	TSE	TSE
Field of view (mm)	200	180	160
Slice thickness (mm)	3	3	3
Matrix size	256 x 256	300 x 300	292 x 276
Pixel size (mm x mm)	0.8 x 0.8	0.6 x 0.6	0.6 x 0.6
TE (ms)	95	100	120
TR (ms)	5340	5407	4815
Acquisition time	4min 2sec	5min 13sec	4min 5 sec
**Diffusion-weighted imaging**			
Sequence	SSEPI	SSEPI	SSEPI
Field of view (mm)	320	220	180
Slice thickness (mm)	5	5	3
Matrix (read)	172 x 172	168 x 168	100 x 100
Pixel size (mm x mm)	1.5 x 1.5	1.29 x 1.29	2.25 x 2.35
TE (ms)	101	80	80
TR (ms)	2200	2304	4391
Multiple *b*-values (s/mm^2)	0, 150, 500, 1000	0, 150, 500, 1000	50, 800
Acquisition time	5min 44sec	6min 15sec	5min 20sec
High *b*-values (s/mm^2)	1400	2000	1600
Acquisition time	3min 39sec	2min6sec	2min42sec
**Dynamic contrast enhanced**			
Sequence	3D FLASH	T1-FFE	T1-TFE
Field of view (mm)	260	180	180
Matrix (read)	192 x 192	140 x 162	140 x 138
Pixel size (mm x mm)	1.4 x 1.4	1.29 x 1.30	1.29 x 1.30
TE (ms)	2.50	2.8	1.83
TR (ms)	5.61	5.8	3.7
Fat suppression	Fat sat	SPAIR	Dixon
Acquisition time	5min 43sec	3min11sec	3min 21sec
Temporal resolution (sec)	13	13	10

Abbreviations: FFE = fast field echo; FLASH = fast low angle shot; OSR = Ospedale San Raffaele; SPAIR = spectral attenuated inversion recovery; SSEPI = single shot echo planar imaging; TE = echo time; TR = repetition time; TSE = Turbo Spin Echo.
